# Inflammatory Bone Marrow Mesenchymal Stem Cells in Multiple Myeloma: Transcriptional Signature and In Vitro Modeling

**DOI:** 10.3390/cancers15215148

**Published:** 2023-10-26

**Authors:** Lei Wang, Weijun Yi, Li Ma, Emily Lecea, Lori A. Hazlehurst, Donald A. Adjeroh, Gangqing Hu

**Affiliations:** 1 Department of Microbiology, Immunology & Cell Biology, West Virginia University, Morgantown, WV 26505, USA; lei.wang1@hsc.wvu.edu (L.W.); wy0003@mix.wvu.edu (W.Y.); li.ma@hsc.wvu.edu (L.M.); eel0009@mix.wvu.edu (E.L.); 2 Lane Department of Computer Science & Electrical Engineering, West Virginia University, Morgantown, WV 26506, USA; donald.adjeroh@mail.wvu.edu; 3 WVU Cancer Institute, West Virginia University, Morgantown, WV 26506, USA; lahazlehurst@hsc.wvu.edu; 4 Department of Pharmaceutical Sciences, School of Pharmacy, West Virginia University, Morganton, WV 26506, USA

**Keywords:** multiple myeloma, bone marrow, inflammatory MSCs, transcriptional signature, IL-1β

## Abstract

**Simple Summary:**

Multiple myeloma cells mainly proliferate in the bone marrow (BM). Mesenchymal stem cells (MSCs) in the BM of MM patients are tumor supportive and exhibit an inflammatory transcription signature and contribute to drug resistance. Due to their rarity in the BM, downstream functional characterization of the cells requires in vitro expansion. We conducted a systemic analysis of public expression data and reported the loss of the inflammatory signature during in vitro expansion. However, further analysis on additional publicly available expression data revealed that cytokine stimulations and coculture with immune cells or cancer cells were able to reactivate the transcription signature. Our findings established a crucial foundation for future research into the contribution of the inflammatory status to the tumor-supportive functions of BM MSCs in disease progression and resistance to therapy.

**Abstract:**

Bone marrow mesenchymal stem cells (BM MSCs) play a tumor-supportive role in promoting drug resistance and disease relapse in multiple myeloma (MM). Recent studies have discovered a sub-population of MSCs, known as inflammatory MSCs (iMSCs), exclusive to the MM BM microenvironment and implicated in drug resistance. Through a sophisticated analysis of public expression data from unexpanded BM MSCs, we uncovered a positive association between iMSC signature expression and minimal residual disease. While in vitro expansion generally results in the loss of the iMSC signature, our meta-analysis of additional public expression data demonstrated that cytokine stimulation, including IL1-β and TNF-α, as well as immune cells such as neutrophils, macrophages, and MM cells, can reactivate the signature expression of iMSCs to varying extents. These findings underscore the importance and potential utility of cytokine stimulation in mimicking the gene expression signature of early passage of iMSCs for functional characterizations of their tumor-supportive roles in MM.

## 1. Introduction

Multiple myeloma (MM) cancer cells mainly proliferate in the bone marrow (BM) and are modulated by mesenchymal stem cells (MSCs) [1,2]. Local immune responses also contribute to tumor progression and resistance to therapy [3]. Targeting immunological dysregulation has significantly enhanced MM treatment outcomes through recent advancements in immunotherapies [4]. However, MM remains a disease characterized by inevitable relapse [5]. Thus, a comprehensive understanding of the BM tumor microenvironment, which includes the interactions among MM cells, stromal cells, and immune cells, is essential for identifying novel therapeutic targets to enhance treatment outcomes.

BM derived MSCs promote MM growth and confer drug resistance [1,2,6,7]. Beyond multilineage differentiation potential [8,9], MSCs secrete soluble factors that regulate the immune microenvironment, augment wound healing, and stimulate angiogenesis [10]. A recent study of BM mononuclear cells has identified a sub-population of MSCs presenting in the MM microenvironment [11]. These cells, termed inflammatory MSCs (iMSCs), produce high levels of MM survival factors (IL6 and LIF), MM recruiting ligand CCL2, and immune cell-attracting chemokines (CXCL2, CXCL3, CXCL5, and CXCL8). Intriguingly, the iMSC phenotype persists after successful anti-myeloma therapy [11], indicating a potential role in promoting drug resistance.

Studies that aim to characterize BM MSCs often require in vitro expansion due to the scarcity of these cells in the BM [11,12,13]. However, in vitro expansion may not precisely mirror the tumor microenvironment, raising concerns about the preservation of MM-specific iMSC signature expressions. Specifically, without the environmental inflammatory cues from the BM, the iMSC signature expression may not persist during in vitro expansion. Intriguingly, cytokine stimulations could enhance immunoregulatory functions of MSCs [14]. We, therefore, wondered whether cytokine stimulations could restore the in vivo iMSC signatures or not. Addressing these questions may help to lay down a solid foundation for an in vitro modeling of iMSCs, supporting future investigation into their roles linked to tumor growth, drug resistance, and immunoregulation.

In this study, we conducted a meta-analysis of public whole-genome gene expression data for BM MSCs from MM patients and healthy donors. The results showed a general decline in the expression of MM-specific iMSC signatures during in vitro expansion. We further investigated the reactivation of the iMSC signature through cytokine stimulation (IL-1β, IFN-γ, TGF-β1, and TNF-α) and coculture with immune cells (neutrophils, macrophages, and T cells). There was a significant activation of the iMSC signature expression with IL-1β and TNF-α stimulations, lesser activation with TGF-β1, but no change with IFN-γ exposure. Neutrophils, macrophages, and T cells also activated the iMSC signature expression, albeit in different gene subsets. Our findings demonstrate the potential of using cytokine stimulation to establish in vitro models of iMSCs and warrant a reevaluation of BM MSC-induced drug resistance by considering their inflammatory status.

## 2. Materials and Methods

### 2.1. Gene Expression Datasets

Single-cell RNA sequencing datasets of FACS isolated BM MSCs of newly diagnosed individuals with MM and healthy donors (HDs) were downloaded from ArrayExpress (E-MTAB-9139) [11]. Gene expression matrices for bulk RNA-sequencing datasets of FACS isolated BM MSCs for HDs and MM patients at diagnosis and after induction therapy were also downloaded (E-MTAB-9285) [11]. For in vitro expanded BM MSCs contrasting MM with HDs, we downloaded gene expression data from GSE113736 [15], GSE137369 [16], GSE146649 [17], GSE46053 [18], GSE78235 [19], GSE36474 [20], GSE80608 [21], GSE196297 [22], and GSE108159 [23] via the Gene Expression Omnibus (GEO). We also obtained gene expression data from GEO or ArrayExpress for HD BM-MSC in vitro expanded and stimulated by various cytokines: GSE129165 by IL-1β, IFN-γ, and TNF-α [24], GSE161762 by IL-1β and TNF-α [25], GSE33755 by IL-1β [26], GSE35331 by TNF-α [27], and GSE77814 by IFN-γ, and TNF-α [28], as well as E-MTAB-5420 by TGF-β1 and E-MTAB-5421 for TNF-α [29]. Gene expression data for HD BM-MSC stimulated by different immune cells were accessible from GEO: GSE62782 by neutrophils [30], GSE75749 by activated T cells, and GSE93970 by macrophages [31]. Lastly, we downloaded from GEO gene expression data for MM/HD BM-MSC cocultured with MM cell line MM.1S (GSE46053) [18] or INA-6 (GSE87073) [32].

### 2.2. Data Analysis

We reanalyzed the single-cell RNA sequencing data generated previously [11] to identify signature genes for inflammatory MSCs from BM of MM patients compared to HDs [11]. Briefly, alignment of the short reads to the human reference genome (hg38) was performed using CellRanger (version 3.0.2, 10× Genomics). Cell type annotation on MSCs from the single-cell data were downloaded from https://github.com/MyelomaRotterdam/de-Jong-et-al.-2021 (accessed on 11 December 2022) [11]. Genes upregulated in BM MSCs of MM compared to HDs were identified using the FindAllMarkers function from Seurat 4.2.1 (Wilcoxon test) with adjusted *p*-value < 0.05 and fold change (FC) > 1.5. Bulk RNA-Seq expression matrix data for E-MTAB-9285 from the same study were downloaded and quantile normalized. Differentially expressed (DE) genes of MM patients vs. HDs were predicted by using *t*-test (*p*-value < 0.05 and FC > 2). To compare MRD positivity or negativity to the same MM patients at diagnosis, we applied paired *t*-test with *p*-value < 0.05 and FC > 2.

For gene set enrichment analysis (GSEA) [33], we constructed GCT and CLS files from the gene expression matrices downloaded from GEO or ArrayExpress. One of the outputs from GSEA includes a so-called “rnk” file, which has gene symbol in the first column and FC of expression in the second column. To compare FC of expression across different studies, quantile normalization was conducted across the “rnk” files. For RNA-seq data where gene expression matrix was not provided, we downloaded the raw sequencing file, applied RNA-Seq pipeline established previously [34], and calculated the FC to construct “rnk” files GSEA. Estimation of the contribution of cytokines in deriving expression changes, measure by CytoSig score, was calculated using the online CytoSig webserver (https://cytosig.ccr.cancer.gov/; accessed on 13 February 2023) [35].

## 3. Results

### 3.1. Transcriptional Signature of Primary MSCs from MM Patients

A recent study by others [11] utilized single-cell RNA-Seq analysis to characterize cellular heterogeneity of BM MSCs sorted from newly diagnosed MM patients. The study found that a sub-population of the cells, termed as inflammatory BM MSCs (iMSCs), exhibit an inflammatory transcriptional signature unique to the MM BM microenvironment. We reanalyzed their single-cell RNA-Seq data and identified 279 upregulated genes in BM MSCs from MM patients as compared to healthy donors (HDs) (Table S1). These genes comprise 82% of the top 50 iMSC-specific genes defined by others [11]. In the rest of the manuscript, we refer to the 279 genes as “iMSC signature genes”.

The previous study [11] also generated bulk-cell RNA-Seq gene expression data for in vivo isolated BM MSCs of MM patients. After downloading and reanalyzing the bulk-cell RNA-Seq data, we identified 667 upregulated and 844 downregulated genes in MM vs. HDs (Figure S1A). Consistent with previous findings [11], common immune-related genes such as *CCL2*, *CD44*, *CXCL3*, *CXCL5*, *CXCL8*, *IL6*, and *LIF* were upregulated in the patient samples (Figure S1A). As expected from scRNA-Seq analysis, the expression of iMSC signature genes was also elevated in the patient samples from the gene set enrichment analysis (GSEA) (Figure S1B). Further GSEA against the MSigDB hallmark gene set collection [36] identified “TNF-α signaling via NF-κB” and “inflammatory response” as top upregulated pathways (Figure S1C). These results reassured the signature expression of iMSC in MM patients and supported bulk-cell RNA-Seq as a reasonable alternative to single-cell RNA-Seq for investigating iMSCs in MM [11].

BM MSCs from MM patients showed impaired differentiation capabilities as compared to those from HDs [12,37]. The RNA-Seq data from de Jong et al. [11] enabled an examination of the underlying molecular pathways through GSEA. Signature genes for adipogenic, osteogenic, and chondrogenic cells in human BM MSCs are from a previous work [38]. The expression of adipogenic signature genes was lower in MM than in HDs (Figure S1D), with examples including *APOD*, a preadipocyte/adipose stem cell marker [39], and *ADIPOQ*, a gene highly expressed in preadipocytes [40]. Similarly, the expression of osteogenic signature genes was downregulated (Figure S1E), including *ALPL*, an early marker of osteogenic differentiation [41], and *COL1A1*/*2*, two pathogenic genes for osteogenesis imperfecta [42]. In contrast, chondrogenic signature genes were upregulated (Figure S1F), including *SOX9* and *SOX6* with known functions in promoting chondrogenesis [43,44]. The results suggested that primary MSCs in MM patients downregulated transcription programs related to adipogenesis and osteogenesis, while upregulating those related to chondrogenesis.

Cancer-associated fibroblast cells (CAFs) play a critical role in solid tumors and a subset displays an inflammatory phenotype [45,46,47,48]. To compare with iMSCs, we collected signature genes for inflammatory CAFs (iCAFs) from single-cell RNA-Seq analysis of pancreatic cancer [45] and colorectal cancer [48]. GSEA revealed that iCAF signature genes were generally overexpressed in the BM MSCs of MM compared to HDs (Figure S1G). Although signature genes shared by iMSCs and iCAFs were enriched in immune-related genes, most of the signature genes were cancer type-specific (Figure S1H). These findings indicate that iMSCs from MM patients are distinct from iCAFs in solid tumors and warrant further investigation.

### 3.2. Expression of iMSC Signature Genes during Minimal Residual Disease

Minimal residual disease (MRD) refers to the presence of cancer cells in complete remission at a rate of no more than 10^−5^ among normal BM cells for MM patients [49]. MRD negativity is a strong prognostic factor for improved outcomes in various MM treatments [50,51]. The work by de Jong et al. [11] generated gene expression data from bulk RNA-Seq analysis for BM MSC samples paired at diagnosis and during MRD. We reanalyzed their expression data to link the expression of iMSC signature genes to MRD status.

**Figure 1:**
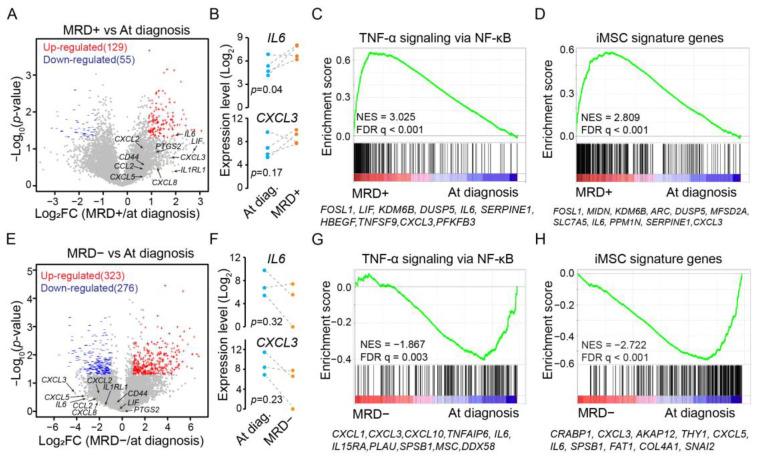
Expression changes in iMSC signature genes associated with MRD status. (**A**) Volcano plot displaying fold changes of expression and *p*-values from bulk-cell RNA-Seq analysis of BM MSCs to differentiate MM samples during MRD positivity from those matched at diagnosis. Red: upregulated genes during MRD positivity. Blue: downregulated genes. Gray: other expressed genes. Indicated by orange dots and arrow heads are examples of immune-related genes. (**B**) Expression level of *IL6* (**upper** panel) and *CXCL3* (**lower** panel) in BM MSCs comparing MM patients during MRD positivity to matched samples at diagnosis, indicated by dot lines. *p*-value by paired *t*-test. (**C**) GSEA of expressed genes sorted by expression changes from high (red) to low (blue) in MSCs collected at MRD+ compared to at diagnosis (calculated from bulk-cell RNA-Seq data) against MSigDB hallmark gene set “TNF-α signaling via NF-κB” (vertical bars). Highlighted are top 10 leading genes. NES: normalized enrichment score. (**D**) GSEA like panel C but against iMSC signature genes defined for MM patients from single-cell RNA-Seq analysis. (**E**) Volcano plot like panel A but comparing BM MSC samples collected during MRD negativity to matched samples at diagnosis. (**F**) Expression level of *IL6* (upper panel) and *CXCL3* (lower panel) comparing MM patients during MRD negativity to those matched at diagnosis. (**G**) GSEA like panel C but comparing BM MSC samples collected during MRD negativity to those matched at diagnosis. (**H**) GSEA like panel D but comparing BM MSC samples collected during MRD negativity to those matched at diagnosis.

We first focused on MM patients with MRD positivity. Two patients were excluded due to high *PTPRC* (CD45) expression, an indication of insufficient depletion of immune cells during MSC isolation. The reanalysis identified hundreds of differentially expressed genes (Figure 1A). Notably, MRD positivity was associated with elevated expression of iMSC signature genes such as *IL6* and *CXCL3* (Figure 1B). At the pathway level, GSEA revealed an overall upregulation in “TNF-α signaling via NF-κB” (Figure 1C). This was further supported by the upregulation of iMSC signature genes (Figure 1D). Therefore, BM MSCs from MRD-positive patients exhibited an enhanced expression of iMSC signatures compared to those at diagnosis.

We then repeated the analysis for patients who achieved MRD negativity after treatment, identifying ~600 differentially expressed genes (Figure 1E). Remarkably, the expression of iMSC signature genes such as *IL6* and *CXCL3* decreased after a full elimination of the cancer cells (Figure 1F). Unlike MRD positivity, “TNF-α signaling via NF-κB” was downregulated during MRD negativity (Figure 1G). As expected, iMSC signature genes were downregulated as well (Figure 1H). Therefore, MRD negativity is concurrent with an attenuated expression of iMSC signature genes in the BM MSCs.

### 3.3. Diminished Expression of iMSC Signature Genes during In Vitro Expansion

MSCs are rare in BM aspirates [11,12]. In vitro expansion is necessary to obtain sufficient cell numbers for downstream functional characterization [12]. Several recent studies have analyzed genome-wide transcriptome for in vitro expanded BM MSCs of MM patients [15,16,17,18,19,20,21,22,23,37,52,53]. We downloaded expression data from these studies if publicly accessible and conducted a meta-analysis to identify molecular pathways specific to BM MSCs in MM patients compared to HD. We excluded studies optimized for long noncoding RNAs [23] or studies with control subjects including other cancers [21,22]. Six studies remained for the meta-analysis [15,16,17,18,19,20] (Table S2). The expression changes were quantile normalized and included in Table S3.

We first checked the expression of several genes coding for soluble factors associated with the pathophysiology of MM, including *IL6*, *DKK1*, and *GDF15*. ELISA results from the literature confirmed the increased protein abundance of the three genes in BM MSCs of MM patients [20,52,54,55]. Consistently, at mRNA levels the expression of the three genes was generally upregulated (Figure S2A).

BM MSCs from MM patients exhibit reduced proliferation compared to healthy MSCs [20,37,54]. We used GSEA to assess the overall expression changes against MSigDB hallmark gene sets [36]. As expected, the analysis revealed a downregulation of cell proliferation-related gene sets in all studies except one (Figure S2B). Examples were shown for G2M checkpoint genes (Figure S2C; first six panels). The expression downregulation of G2M checkpoint genes was further confirmed by comparing to house-keeping genes (Figure S2D). In contrast, G2M checkpoint genes were upregulated in MSCs isolated from MM patients without in vitro expansion (Figure S2C; bottom panel). Thus, in vitro expansion resulted in a downregulation of proliferation-related genes in contrast to the observation made with in vivo BM MSCs.

**Figure 2:**
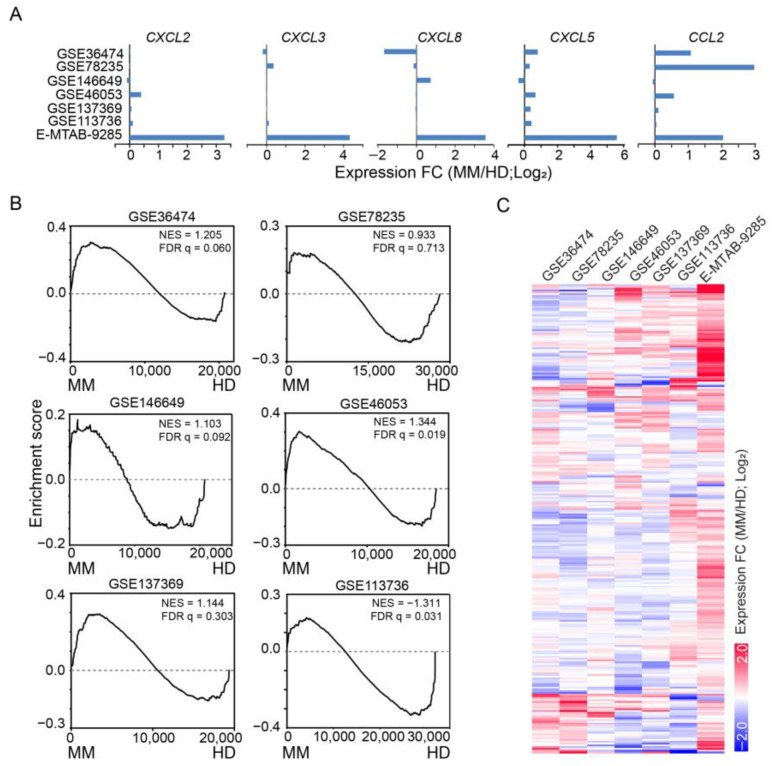
Loss of expression upregulation in inflammatory genes in expanded MM MSCs. (**A**) Fold change of expression (MM/HD) for inflammatory genes highlighted in de Jong, et al. [11] from studies based on expanded MSCs (GSE#s) or sorted primary MSCs (E-MTAB-9285). (**B**) GSEA of expressed genes sorted by expression changes in in vitro expanded MSCs (MM/HD) from high (**left**) to low (**right**) against iMSC signature genes. NES: normalized enrichment score. GEO accession numbers indicated for each study. (**C**) Heatmap visualization of expression fold change (MM/HD) for iMSC signature genes (rows) across studies (columns).

In vivo BM MSCs from MM patients exhibit high expression of inflammatory genes such as *CCL2*, *CXCL2*, *CXCL3*, *CXCL5*, *CXCL8*, and *PTGS2* [11]. However, the expression upregulation of the immune-related genes was generally lost during in vitro expansion (Figure 2A). Consistently, an overall increase or decrease in the expression of iMSC signature genes from GSEA was not observed for in vitro expanded cells (Figure 2B). Visual inspection of the expression changes by heatmap further supported this conclusion (Figure 2C). Therefore, MSCs expanded in vitro lost the patient-specific expression of iMSC signature genes in the absence of the BM microenvironment.

### 3.4. Expression Activation of iMSC Signature Genes by Cytokine Stimulation

In addition to self-renewal and multipotency, MSCs exhibits immune regulatory functions. Common factors impacting the immunomodulatory potential of MSCs are IL-1β, IFN-γ, TGF-β, and TNF-α [14]. A survey of public expression data was conducted on BM MSCs stimulated with various cytokines to determine their potential in activating the iMSC signature expression in vitro. Six studies were identified [24,25,26,27,28,29] (Table S4). Expression changes induced by these stimulations were compiled and quantile normalized (Table S5).

**Figure 3:**
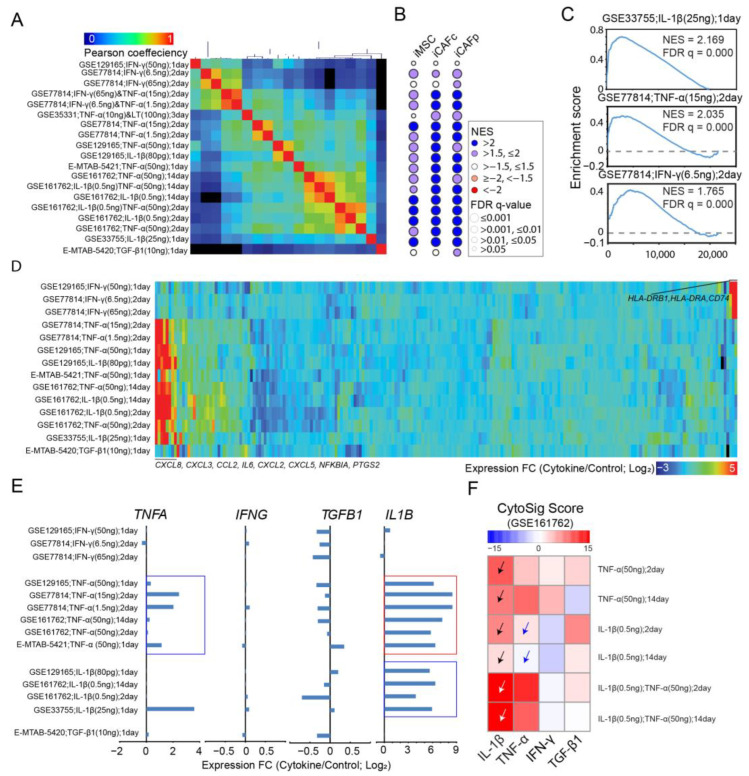
Expression activation of iMSC signature genes by cytokines. (**A**) Hierarchy clustering analysis for cytokine(s) based on Pearson correlation of genome-wide expression changes induced by the stimulations. Indicated are cytokine, doses, and durations from different studies with accession numbers. (**B**) Bubble plots for NES and FDR q values of GSEA applied to genes sorted by expression changes induced by cytokines against signature genes of iMSCs in MM patients (**leftmost** column) and iCAFs in colorectal cancer patients (**middle** column) or pancreatic cancer patients (**rightmost** column). Each row represents a cytokine stimulation with annotation aligned with panel A. (**C**) Representative results for GSEA from panel B for stimulations by IL1-β (**top** panel), TNF-α (**middle** panel), and IFN-γ (**bottom** panel). (**D**) Heatmap visualization of expression changes induced by mono-treatment of cytokines such as IFN-γ, IL1-β, TNA-α, and TGF-β for iMSC signature genes defined for MM patients. (**E**) Bar plots for expression changes of *TNFA*, *IFNG*, *TGFB1*, and *IL1B* in BM MSCs stimulated by IL1-β, IFN-γ, TNF-α, or TGF-β1. (**F**) Heatmap visualization of CytoSig score, which predicts cellular response to cytokines (columns), based on expression changes induced by external stimulations (rows). Black arrow heads: cellular response to IL-1β was upregulated when stimulated by TNF-α or IL-1β; White arrow heads: cellular response to IL-1β was further upregulated when co-stimulated by TNF-α and IL-1β; Blue arrow heads: cellular response to TNF-α was not activated when stimulated by IL-1β.

Hierarchical clustering analysis based on correlations of expression changes segregated the samples into two major clades corresponding to the use of IFN-γ or others, and then further into sub-clades coincident with the studies (Figure 3A). GSEA revealed an overall upregulation in immune function-related gene sets such as “TNF-α signaling via NF-κB”, “IFN-γ response” (with the exception to TGF-β1 stimulation), and “inflammatory response” regardless of cytokine use, doses, and duration of the stimuli (Figure S3A). To minimize confounding effects in results interpretation, we narrowed down to mono-stimulations. Gene clustering analysis based on expression changes within the gene set “TNF-a signaling through NF-κB” revealed more dramatic expression upregulation by TNF-α or IL-1β than by IFN-γ or TGF-β1 (Figure S3B). Similar analysis of IFN-γ response (Figure S3C) and inflammatory response (Figure S3D) revealed preferential activation of subsets of genes within each gene set by different cytokines. Additionally, expression changes were generally similar between the stimulations of TNF-α and IL-1β, especially for “TNF-a signaling through NF-κB” (Figure S3B–D).

We next examined the impact of cytokines on the expression of iMSC signature genes. GSEA showed an overall upregulation of these genes under most cytokine stimulations, except for TGF-β1 and a high dose of IFN-γ (Figure 3B), with examples from specific studies shown in Figure 3C. Similar observation was made for iCAF signature genes (Figure 3B). A heatmap analysis of transcription responses to mono-cytokine confirmed the overall upregulation of iMSC signature genes, particularly for immune-related genes such as *CCL2*, *CXCL3*, *CXCL5*, *CXCL8*, *IL6*, and *PTGS2*, upon TNF-a or IL-1β stimulation (Figure 3D). To determine the interdependence between cytokines, we analyzed expression changes for genes *IL1B*, *IFNG*, *TGFB1*, and *TNF* (Figure 3E). The results showed that *INFG* and *TGFB1* remained insensitive to stimulations, while *TNF* and *IL1B* were upregulated by their corresponding cytokines (Figure 3E; blue rectangles). Intriguingly, TNF-α substantially activated the transcription level of *IL1B* (Figure 3E; red rectangle), suggesting a role of IL-1β in the transcription response to TNF-α. Indeed, CytoSig cytokine response analysis [35] revealed that cellular response to IL-1β was upregulated when stimulated by TNF-α or IL-1β (Figure 3F; black arrow heads) and was further elevated by combined stimulations (Figure 3F; white arrow heads). In contrast, IL-1β did not activate or only modestly activated the cellular response to TNF-α (Figure 3F; blue arrow heads). These findings highlighted the complex and dynamic nature of cytokine-induced transcription regulation in BM MSCs.

### 3.5. Expression Activation of iMSC Signature Genes by Immune Cells

**Figure 4:**
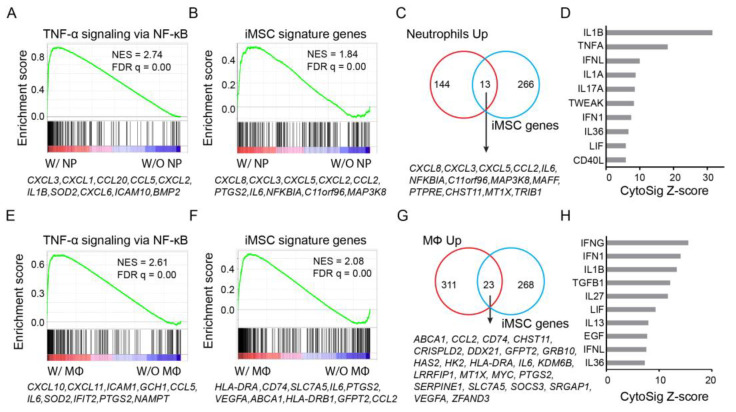
Expression activation of iMSC signature genes with neutrophils or macrophages. (**A**) GSEA of expressed genes sorted by expression changes in BM MSCs induced by neutrophils (NP) from high (red) to low (blue) against hallmark gene set “TNF-α signaling via NF-κB” (vertical bars). Highlighted are top 10 leading genes. NES: normalized enrichment score. (**B**) Like panel A, but against iMSC signature genes for MM patients. (**C**) Venn diagram for genes upregulated by neutrophils and iMSC signature genes. (**D**) Bar plot for CytoSig score, which predicts cytokines contributing to the expression changes in BM MSCs as induced by neutrophils. (**E**) GSEA of expressed genes sorted by expression changes of BM MSCs induced by inflammatory macrophages (MΦ) from high (red) to low (blue) against hallmark gene set “TNF-α signaling via NF-κB” (vertical bars). Highlighted are top 10 leading genes. (**F**) Like panel E, but against iMSC signature genes. (**G**) Venn diagram for genes upregulated by inflammatory macrophages and iMSC signature genes. (**H**) Bar plot for CytoSig score, which predicts cytokines contributing to the expression changes in BM MSCs as induced by inflammatory macrophages.

MSCs are known as a sensor of the inflammatory environment through a tight interaction with immune cells. BM MSCs from MM patients secrete a high level of CXCR1/CXCR2 ligands such as CXCL2, CXCL3, CXCL5, and IL-8 (CXCL8) [11], which attract neutrophils, monocytes, and natural killer cells [56]. We next explored the possibility of activating the iMSC signature expression in BM MSCs with neutrophils or macrophages (Table S6). The expression data generated by Gregoire et al. [30] allows us to address the possibility for neutrophils. We downloaded their expression data generated for BM MSCs cocultured with neutrophils. Our reanalysis revealed that “TNF-α signaling via NF-κB” (Figure 4A) and iMSC signature genes (Figure 4B) were upregulated in the presence of neutrophils. Immune-related genes significantly upregulated by neutrophils included iMSC signature genes such as *CCL2*, *CXCL3*, *CXCL5*, *CXCL8*, and *IL6* (Figure 4C). CytoSig analysis ranked IL-1β and TNF-α as the top two cytokines driving the expression changes induced by neutrophils in BM MSCs (Figure 4D).

Espagnolle et al. [31] generated expression data for BM MSCs cocultured with pro-inflammatory or anti-inflammatory macrophages. Consistent with previous findings [31], coculture with anti-inflammatory macrophages induced a minimal level of expression changes in MSCs. In contrast, the presence of pro-inflammatory macrophages upregulated the expression of “TNF-α signaling via NF-κB” (Figure 4E) and iMSC signature gene (Figure 4F). However, pro-inflammatory macrophages failed to upregulate the expression of CXCR1/CXCR2 ligand genes such as *CXCL2*, *CXCL3*, *CXCL5*, and *CXCL8* (Figure 4G). CytoSig analysis indicated IFN-γ and IFN1 as the top two cytokines driving the expression changes (Figure 4H). Consistently with the results from in vitro stimulation by IFN-γ (Figure 3D), *CD74*, *HLA-DRA*, and *HLA-DRB1* are among the leading genes accounting for the most expression changes in iMSC signatures induced by pro-inflammatory macrophages (Figure 4F).

Differentially expressed genes in BM MSCs in response to CD3/CD28 activated T cells in a mouse model are publicly available in the GEO database (accession number GSE75749). We identified 649 genes upregulated by activated T cells (fold change > 2 and adjusted *p*-value < 0.05): enrichment analysis by Metascape [57] revealed that these genes were linked to functions linked to “INF-γ response” and “TNF-α signaling via NF-κB” (Figure S4A). Activated T cells upregulated 36.5% and 17.5% of the genes in the two gene sets, respectively (Figure S4B). GSEA demonstrated that the 649 upregulated genes were also upregulated in the BM MSCs of MM patients when compared to HDs (Figure S4C). We observed similar upregulation after excluding genes annotated with either “INF-γ response” or “TNF-α signaling via NF-κB” (Figure S4D), suggesting additional pathways relevant to the iMSC signature activation. In fact, leading-edge genes in Figure S4D were enriched in iMSC-relevant gene sets, such as “TGF-β signaling” and “UV response downregulation” (Figures S1C and S4E).

### 3.6. Expression Activation of iMSC Signature Genes by Multiple Myeloma Cells

Genome-wide expression profiling of BM MSCs cocultured with MM cancer cells has been previously conducted [18,32]. Garcia-Gomez et al. [18] cocultured BM MSCs from HDs or MM patients with the MM.1S cell line and compared to a monoculture of BM MSCs. Similarly, Dotterweich et al. [32] cocultured BM MSCs with the INA-6 cell line. We reanalyzed the expression data from both studies to investigate the impacts of MM cells on the expression of iMSC signature genes in BM MSCs.

**Figure 5:**
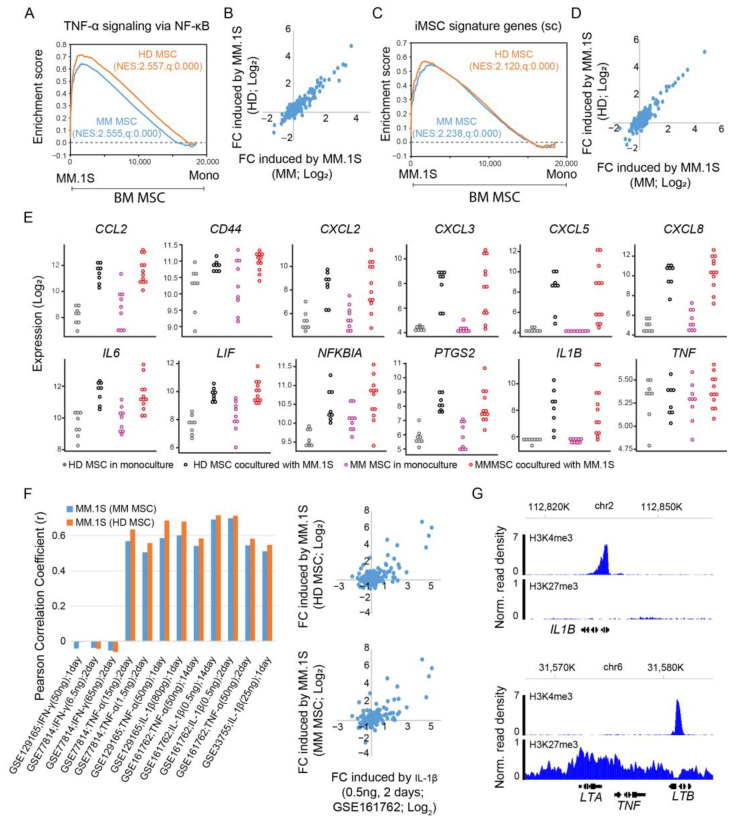
Expression activation of iMSC signature genes by MM.1S. (**A**) GSEA of expressed genes sorted by expression changes induced by MM.1S (as compared to monoculture; Mono) in BM MSCs of MM patients (MM; red line) or healthy donors (HD; black line) from high (**left** side) to low (**right** side) against MSigDB hallmark gene set “TNF-α signaling via NF-κB”. NES: normalized enrichment score. (**B**) Scatter plot comparing the expression changes in genes from “TNF-α signaling via NF-κB” induced by MM.1S in BM MSCs from MM (*x*-axis) to those from HDs (*y*-axis). (**C**) Like panel A, but against iMSC signature genes. (**D**) Like panel B, but for iMSC signature genes. (**E**) Dot plots for the expression of immune-related genes in BM MSCs from MM and HDs in coculture with MM.1S or in monoculture. (**F**) Pearson correlation measuring the similarity of expression changes induced by MM.1S (in HD MSCs and MM MSCs) and those induced by cytokines, with an example comparing a stimulation by MM.1S to by IL1-β. (**G**) Normalized ChIP-seq read density for histone post-translational modifications H3K4me3 and H3K27me3 across gene locus IL1B (**upper** panel) and TNF (**lower** panel) in BM MSCs.

In the reanalysis of expression data generated by Garcia-Gomez et al. [18], we observed an overall upregulation of “TNF-α signaling via NF-κB” in MM.1S-cocultured BM MSCs from both MM patients and healthy donors as compared to monoculture (Figure 5A). MM.1S induced expression changes in this pathway were highly correlated between MM patients and HDs (Figure 5B). Likewise, MM.1S upregulated the expression of iMSCs signature genes (Figure 5C), with consistent expression changes between MM and HDs (Figure 5D). We next explored the impact of MM cells on the expression of immune-related genes in BMSCs. For monocultured BM MSCs, the expression of *CCL2*, *CXCL2*, *IL6*, and *LIF* was slightly higher in MM patients compared to healthy donors (pink vs. gray). In coculture with MM.1S, expression of the immune-related genes increased to comparable levels between MM and HDs (red vs. black) (Figure 5E).

The MM.1S-induced expression changes in iMSC signature genes demonstrated a positive correlation with those stimulated by cytokines, such as IL-1β and TNF-α, but not IFN-γ (Figure 5F). Intriguingly, IL1B expression in MSCs was substantially upregulated by MM.1S, while *TNF* expression remained unaltered (Figure 5E). In line with this observation, we analyzed public ChIP-Seq data for BM MSCs [58]. We observed the presence of active histone marker H3K4me3 and the absence of repressive histone marker H3K27me3 at the *IL1B* promoter region, meaning that *IL1B* is primed for transcriptional activation upon external stimuli. In contrast, the *TNF* promoter displayed an opposite chromatin configuration (Figure 5G). Thus, MM.1S may activate inflammatory signature genes in MSCs through the activation of *IL1B* expression.

Lastly, we reanalyzed expression data from BM MSCs cocultured with the IL-6 dependent MM cell line, INA-6, and in monoculture [32]. GSEA revealed no significant upregulation in the “TNF-α signaling via NF-κB hallmark” gene set (Figure S5A) or in the iMSC signature genes (Figure S5B). In line with these findings, a close examination of individual immune-related genes revealed minimal expression changes, with only a few exceptions (Figure S5C). Thus, unlike MM.1S, INA-6 did not upregulate the iMSC signature expression.

## 4. Discussion

Mesenchymal stem cells (MSCs) play an important role in supporting MM cells in the BM microenvironments [1,2]. Intriguingly, BM inflammatory MSCs (iMSCs) are unique to MM patients compared to healthy donors [11]. Their transcription signatures can be robustly identified using both single-cell and bulk-cell RNA-Seq analysis [11,12]. Our data reanalysis identified additional features of iMSCs, such as downregulation of adipogenic or osteogenic genes, upregulation of chondrogenic genes, and upregulation of signature genes corresponding to inflammatory CAF cells (iCAFs) identified from solid tumors. Our reanalysis further showed that at gene set level, the expression of iMSC signature genes was upregulated during MRD positivity and downregulated during MRD negativity. This reenforced presence of iMSCs during MRD positivity implied a role in drug resistance and disease relapse.

Considering that MSCs are scarce in bone marrow aspirates, they are often expanded in vitro for downstream functional characterizations. Once removed from the MM bone marrow microenvironment, MSCs are expected to lose the expression of iMSC signature genes during regular in vitro expansion due to the absence of proinflammatory signals. This prediction was confirmed by our meta-analysis of expression data from BM MSCs expanded from MM patients and controls across multiple studies.

TNF-α and IL-1β are the two major cytokines predicted to drive the observed inflammatory transcriptional signatures in BM MSCs of MM patients [11]. We confirmed this observation by assessing the transcriptional response of bone marrow MSCs to these two cytokines or their combinations using expression data from other studies [24,25,26,28,29]. These findings suggest activation of the NF-κB pathway possibly by TNF-α or IL-1β as a potential target to normalize iMSCs [11,59]. In contrast, stimulation with TGF-β1 and high doses of IFN-γ failed to activate the iMSC signatures. Interestingly, TNF-α stimulation upregulated the mRNA expression of *IL1B* but the inverse was not true. Further experiments with IL1B knockdown or knockout are necessary to clarify the potential dependency on *IL1B* activation for the transcriptional response to TNF-α.

The secretion of CXCR1/CXCR2 ligands by BM MSCs from MM patients [11] attracts neutrophils and monocytes [56]. Our data analysis indicated that the presence of neutrophils increased the expression of CXC1/CXCR2 ligand genes in BM MSCs, potentializing a positive feedback loop for neutrophil recruitment and stromal cell activations [60]. Unlike neutrophils, pro-inflammatory macrophages activated a different subset of iMSC signature genes, including those responsive to IFN-γ stimulation. Similar observations were made for BMSCs when stimulated with CD3/CD28 activated T cells.

BM MSCs promote drug resistance in MM cells through the secretion of soluble factors and physical interaction [2,61]. The persistent presence of iMSCs in MRD suggests a link to drug resistance and disease relapse. Similarly, iCAFs in solid tumors enhance therapy resistance [48]. Interestingly, the presence of MM cells activated the iMSC signature in BMSCs, although this varied with cancer subtypes. As exemplified with MM.1S, the presence of MM cells upregulated the expression of *IL1B* other than *TNF* in the BM MSCs, indicating a differential role of IL-1β in activating the iMSC signature expression. Considering the connection of iMSCs with MRD, it is worth reevaluating the role of BM stroma-induced drug resistance in MM cells by considering the contribution from the inflammatory state in the MSCs. Equally important is to examine their roles in modulating the immune microenvironment, as recently demonstrated in the iMSC-specific activation of neutrophils [62]. Establishing an in vitro model for iMSCs through cytokine stimulations such as IL-1β represents an important step to address this issue. Further research into the role of iMSCs in modulating drug resistance should clearly differentiate the effects of cytokine-induced iMSCs from the cytokines themselves. For example, while it is common to prime MSCs with cytokines and then remove the cytokine from the culture condition, MM cells stimulated by the cytokines provide an essential control to account for any residual cytokines from the priming process. In addition to drug resistance, BM MSCs protect MM cells from CAR T cell-mediated cytotoxicity [63]. While other studies, such as the one by Dhodapkar, et al. [64], have explored the effects of CART therapy on the BM tumor/immune cells from MM patients, they have not specifically focused on BM MSCs. As a result, the exact influence of iMSCs on CART T therapy, and vice versa, remains an open question that warrants further investigation.

## 5. Conclusions

Our work has systematically characterized the molecular signatures of iMSCs in MM patients and confirmed a general loss of this signature during in vitro expansion. Importantly, we observed a robust activation of iMSC signature expression in BM MSCs with IL-1β and/or TNF-α stimulation, as well as coculture with neutrophils. Our work represents a significant advancement towards the establishment of in vitro models for iMSCs and future work to understand their role in BM MSC-induced drug resistance in MM cells.

## Data Availability

The data presented in this study are available in this article.

## Supplementary Materials

The following supporting information can be downloaded at: https://www.mdpi.com/article/10.3390/cancers15215148/s1, Figure S1: Molecular signatures of iMSCs captured by bulk cell RNA-Seq for MM patients; Figure S2: Downregulation of proliferation-related pathways in MM MSCs expanded in vitro; Figure S3: Expression activation of TNF-α signaling pathway in MM MSCs by cytokines; Figure S4: Expression activation of iMSC signature genes by activated T cells; Figure S5: INA-6 failed to active the expression of iMSC signature genes; Table S1: List of iMSC signature genes; Table S2: List of expression data for BM MSCs from MM patients and healthy donors; Table S3: Fold-change of expression (patient/healthy donors; quantile normalized) compiled from six studies; Table S4: List of expression data for BM MSCs stimulated with various cytokines; Table S5: Fold-change of expression (stimulation/non-stimulation; quantile normalized) compiled from six studies that stimulate BMSCs with different cytokines; Table S6: List of expression data for BM MSCs cocultured with immune cells.

## References

[1] Maiso P., Mogollon P., Ocio E.M., Garayoa M. (2021). Bone Marrow Mesenchymal Stromal Cells in Multiple Myeloma: Their Role as Active Contributors to Myeloma Progression. Cancers.

[2] Chen W.C., Hu G., Hazlehurst L.A. (2020). Contribution of the bone marrow stromal cells in mediating drug resistance in hematopoietic tumors. Curr. Opin. Pharmacol..

[3] Diakos C.I., Charles K.A., McMillan D.C., Clarke S.J. (2014). Cancer-related inflammation and treatment effectiveness. Lancet Oncol..

[4] Pinto V., Bergantim R., Caires H.R., Seca H., Guimaraes J.E., Vasconcelos M.H. (2020). Multiple Myeloma: Available Therapies and Causes of Drug Resistance. Cancers.

[5] Kumar S., Baizer L., Callander N.S., Giralt S.A., Hillengass J., Freidlin B., Hoering A., Richardson P.G., Schwartz E.I., Reiman A. (2022). Gaps and opportunities in the treatment of relapsed-refractory multiple myeloma: Consensus recommendations of the NCI Multiple Myeloma Steering Committee. Blood Cancer J..

[6] Filippi I., Saltarella I., Aldinucci C., Carraro F., Ria R., Vacca A., Naldini A. (2018). Different Adaptive Responses to Hypoxia in Normal and Multiple Myeloma Endothelial Cells. Cell. Physiol. Biochem..

[7] Reagan M.R., Ghobrial I.M. (2012). Multiple myeloma mesenchymal stem cells: Characterization, origin, and tumor-promoting effects. Clin. Cancer Res..

[8] Russell K.C., Phinney D.G., Lacey M.R., Barrilleaux B.L., Meyertholen K.E., O’Connor K.C. (2010). In vitro high-capacity assay to quantify the clonal heterogeneity in trilineage potential of mesenchymal stem cells reveals a complex hierarchy of lineage commitment. Stem Cells.

[9] Banfi A., Muraglia A., Dozin B., Mastrogiacomo M., Cancedda R., Quarto R. (2000). Proliferation kinetics and differentiation potential of ex vivo expanded human bone marrow stromal cells: Implications for their use in cell therapy. Exp. Hematol..

[10] Prockop D.J., Oh J.Y. (2012). Mesenchymal Stem/Stromal Cells (MSCs): Role as Guardians of Inflammation. Mol. Ther..

[11] De Jong M.M.E., Kellermayer Z., Papazian N., Tahri S., Hofste Op Bruinink D., Hoogenboezem R., Sanders M.A., van de Woestijne P.C., Bos P.K., Khandanpour C. (2021). The multiple myeloma microenvironment is defined by an inflammatory stromal cell landscape. Nat. Immunol..

[12] Alameda D., Saez B., Lara-Astiaso D., Sarvide S., Lasa M., Alignani D., Rodriguez I., Garate S., Vilas A., Paiva B. (2020). Characterization of freshly isolated bone marrow mesenchymal stromal cells from healthy donors and patients with multiple myeloma: Transcriptional modulation of the microenvironment. Haematologica.

[13] Sklavenitis-Pistofidis R., Haradhvala N.J., Getz G., Ghobrial I.M. (2021). Inflammatory stromal cells in the myeloma microenvironment. Nat. Immunol..

[14] Sarsenova M., Kim Y., Raziyeva K., Kazybay B., Ogay V., Saparov A. (2022). Recent advances to enhance the immunomodulatory potential of mesenchymal stem cells. Front. Immunol..

[15] Fernando R.C., Mazzotti D.R., Azevedo H., Sandes A.F., Rizzatti E.G., de Oliveira M.B., Alves V.L.F., Eugenio A.I.P., de Carvalho F., Dalboni M.A. (2019). Transcriptome Analysis of Mesenchymal Stem Cells from Multiple Myeloma Patients Reveals Downregulation of Genes Involved in Cell Cycle Progression, Immune Response, and Bone Metabolism. Sci. Rep..

[16] Garcia-Gomez A., Li T.L., de la Calle-Fabregat C., Rodriguez-Ubreva J., Ciudad L., Catala-Moll F., Godoy-Tena G., Martin-Sanchez M., San-Segundo L., Muntion S. (2021). Targeting aberrant DNA methylation in mesenchymal stromal cells as a treatment for myeloma bone disease. Nat. Commun..

[17] Lemaitre L., DoSouto Ferreira L., Joubert M.V., Avet-Loiseau H., Martinet L., Corre J., Couderc B. (2020). Imprinting of Mesenchymal Stromal Cell Transcriptome Persists even after Treatment in Patients with Multiple Myeloma. Int. J. Mol. Sci..

[18] Garcia-Gomez A., De Las Rivas J., Ocio E.M., Diaz-Rodriguez E., Montero J.C., Martin M., Blanco J.F., Sanchez-Guijo F.M., Pandiella A., San Miguel J.F. (2014). Transcriptomic profile induced in bone marrow mesenchymal stromal cells after interaction with multiple myeloma cells: Implications in myeloma progression and myeloma bone disease. Oncotarget.

[19] Umezu T., Imanishi S., Azuma K., Kobayashi C., Yoshizawa S., Ohyashiki K., Ohyashiki J.H. (2017). Replenishing exosomes from older bone marrow stromal cells with miR-340 inhibits myeloma-related angiogenesis. Blood Adv..

[20] Andre T., Meuleman N., Stamatopoulos B., De Bruyn C., Pieters K., Bron D., Lagneaux L. (2013). Evidences of early senescence in multiple myeloma bone marrow mesenchymal stromal cells. PLoS ONE.

[21] McNee G., Eales K.L., Wei W., Williams D.S., Barkhuizen A., Bartlett D.B., Essex S., Anandram S., Filer A., Moss P.A. (2017). Citrullination of histone H3 drives IL-6 production by bone marrow mesenchymal stem cells in MGUS and multiple myeloma. Leukemia.

[22] Heinemann L., Mollers K.M., Ahmed H.M.M., Wei L., Sun K., Nimmagadda S.C., Frank D., Baumann A., Poos A.M., Dugas M. (2022). Inhibiting PI3K-AKT-mTOR Signaling in Multiple Myeloma-Associated Mesenchymal Stem Cells Impedes the Proliferation of Multiple Myeloma Cells. Front. Oncol..

[23] Li B., Xu H., Han H., Song S., Zhang X., Ouyang L., Qian C., Hong Y., Qiu Y., Zhou W. (2018). Exosome-mediated transfer of lncRUNX2-AS1 from multiple myeloma cells to MSCs contributes to osteogenesis. Oncogene.

[24] Wiese D.M., Wood C.A., Ford B.N., Braid L.R. (2022). Cytokine Activation Reveals Tissue-Imprinted Gene Profiles of Mesenchymal Stromal Cells. Front. Immunol..

[25] Rubinstein-Achiasaf L., Morein D., Ben-Yaakov H., Liubomirski Y., Meshel T., Elbaz E., Dorot O., Pichinuk E., Gershovits M., Weil M. (2021). Persistent Inflammatory Stimulation Drives the Conversion of MSCs to Inflammatory CAFs That Promote Pro-Metastatic Characteristics in Breast Cancer Cells. Cancers.

[26] Carrero R., Cerrada I., Lledo E., Dopazo J., Garcia-Garcia F., Rubio M.P., Trigueros C., Dorronsoro A., Ruiz-Sauri A., Montero J.A. (2012). IL1beta induces mesenchymal stem cells migration and leucocyte chemotaxis through NF-kappaB. Stem Cell Rev. Rep..

[27] Guilloton F., Caron G., Menard C., Pangault C., Ame-Thomas P., Dulong J., De Vos J., Rossille D., Henry C., Lamy T. (2012). Mesenchymal stromal cells orchestrate follicular lymphoma cell niche through the CCL2-dependent recruitment and polarization of monocytes. Blood.

[28] Jin P., Zhao Y., Liu H., Chen J., Ren J., Jin J., Bedognetti D., Liu S., Wang E., Marincola F. (2016). Interferon-gamma and Tumor Necrosis Factor-alpha Polarize Bone Marrow Stromal Cells Uniformly to a Th1 Phenotype. Sci. Rep..

[29] Lerrer S., Liubomirski Y., Bott A., Abnaof K., Oren N., Yousaf A., Korner C., Meshel T., Wiemann S., Ben-Baruch A. (2017). Co-Inflammatory Roles of TGFbeta1 in the Presence of TNFalpha Drive a Pro-inflammatory Fate in Mesenchymal Stem Cells. Front. Immunol..

[30] Gregoire M., Guilloton F., Pangault C., Mourcin F., Sok P., Latour M., Ame-Thomas P., Flecher E., Fest T., Tarte K. (2015). Neutrophils trigger a NF-kappaB dependent polarization of tumor-supportive stromal cells in germinal center B-cell lymphomas. Oncotarget.

[31] Espagnolle N., Balguerie A., Arnaud E., Sensebe L., Varin A. (2017). CD54-Mediated Interaction with Pro-inflammatory Macrophages Increases the Immunosuppressive Function of Human Mesenchymal Stromal Cells. Stem Cell Rep..

[32] Dotterweich J., Schlegelmilch K., Keller A., Geyer B., Schneider D., Zeck S., Tower R.J., Ebert R., Jakob F., Schutze N. (2016). Contact of myeloma cells induces a characteristic transcriptome signature in skeletal precursor cells -Implications for myeloma bone disease. Bone.

[33] Subramanian A., Tamayo P., Mootha V.K., Mukherjee S., Ebert B.L., Gillette M.A., Paulovich A., Pomeroy S.L., Golub T.R., Lander E.S. (2005). Gene set enrichment analysis: A knowledge-based approach for interpreting genome-wide expression profiles. Proc. Natl. Acad. Sci. USA.

[34] Dziadowicz S., Wang L., Akhter H., Aesoph D., Sharma T., Adjeroh D., Hazlehurst L., Hu G. (2022). Bone Marrow Stroma-induced Transcriptome and Regulome Signatures of Multiple Myeloma. Cancers.

[35] Jiang P., Zhang Y., Ru B.B., Yang Y., Vu T., Paul R., Mirza A., Altan-Bonnet G., Liu L.R., Ruppin E. (2021). Systematic investigation of cytokine signaling activity at the tissue and single-cell levels. Nat. Methods.

[36] Liberzon A., Birger C., Thorvaldsdottir H., Ghandi M., Mesirov J.P., Tamayo P. (2015). The Molecular Signatures Database (MSigDB) hallmark gene set collection. Cell Syst..

[37] Choi H., Kim Y., Kang D., Kwon A., Kim J., Kim J.M., Park S.S., Kim Y.J., Min C.K., Kim M. (2020). Common and different alterations of bone marrow mesenchymal stromal cells in myelodysplastic syndrome and multiple myeloma. Cell Proliferat..

[38] Wang Z., Li X., Yang J., Gong Y., Zhang H., Qiu X., Liu Y., Zhou C., Chen Y., Greenbaum J. (2021). Single-cell RNA sequencing deconvolutes the in vivo heterogeneity of human bone marrow-derived mesenchymal stem cells. Int. J. Biol. Sci..

[39] Vijay J., Gauthier M.F., Biswell R.L., Louiselle D.A., Johnston J.J., Cheung W.A., Belden B., Pramatarova A., Biertho L., Gibson M. (2020). Single-cell analysis of human adipose tissue identifies depot- and disease-specific cell types. Nat. Metab..

[40] Lara-Castro C., Fu Y., Chung B.H., Garvey W.T. (2007). Adiponectin and the metabolic syndrome: Mechanisms mediating risk for metabolic and cardiovascular disease. Curr. Opin. Lipidol..

[41] Li N., Zhou L., Xie W.L., Zeng D.Y., Cai D.Q., Wang H.Y., Zhou C.R., Wang J., Li L.H. (2019). Alkaline phosphatase enzyme-induced biomineralization of chitosan scaffolds with enhanced osteogenesis for bone tissue engineering. Chem. Eng. J..

[42] Zhytnik L., Maasalu K., Pashenko A., Khmyzov S., Reimann E., Prans E., Koks S., Martson A. (2019). COL1A1/2 Pathogenic Variants and Phenotype Characteristics in Ukrainian Osteogenesis Imperfecta Patients. Front. Genet..

[43] Akiyama H., Kim J.E., Nakashima K., Balmes G., Iwai N., Deng J.M., Zhang Z., Martin J.F., Behringer R.R., Nakamura T. (2005). Osteo-chondroprogenitor cells are derived from Sox9 expressing precursors. Proc. Natl. Acad. Sci. USA.

[44] Liu C.F., Lefebvre V. (2015). The transcription factors SOX9 and SOX5/SOX6 cooperate genome-wide through super-enhancers to drive chondrogenesis. Nucleic Acids Res..

[45] Ohlund D., Handly-Santana A., Biffi G., Elyada E., Almeida A.S., Ponz-Sarvise M., Corbo V., Oni T.E., Hearn S.A., Lee E.J. (2017). Distinct populations of inflammatory fibroblasts and myofibroblasts in pancreatic cancer. J. Exp. Med..

[46] Elyada E., Bolisetty M., Laise P., Flynn W.F., Courtois E.T., Burkhart R.A., Teinor J.A., Belleau P., Biffi G., Lucito M.S. (2019). Cross-Species Single-Cell Analysis of Pancreatic Ductal Adenocarcinoma Reveals Antigen-Presenting Cancer-Associated Fibroblasts. Cancer Discov..

[47] Kieffer Y., Hocine H.R., Gentric G., Pelon F., Bernard C., Bourachot B., Lameiras S., Albergante L., Bonneau C., Guyard A. (2020). Single-Cell Analysis Reveals Fibroblast Clusters Linked to Immunotherapy Resistance in Cancer. Cancer Discov..

[48] Nicolas A.M., Pesic M., Engel E., Ziegler P.K., Diefenhardt M., Kennel K.B., Buettner F., Conche C., Petrocelli V., Elwakeel E. (2022). Inflammatory fibroblasts mediate resistance to neoadjuvant therapy in rectal cancer. Cancer Cell.

[49] Kumar S., Paiva B., Anderson K.C., Durie B., Landgren O., Moreau P., Munshi N., Lonial S., Blade J., Mateos M.V. (2016). International Myeloma Working Group consensus criteria for response and minimal residual disease assessment in multiple myeloma. Lancet Oncol..

[50] Ding H., Xu J., Lin Z., Huang J., Wang F., Yang Y., Cui Y., Luo H., Gao Y., Zhai X. (2021). Minimal residual disease in multiple myeloma: Current status. Biomark. Res..

[51] Munshi N.C., Avet-Loiseau H., Anderson K.C., Neri P., Paiva B., Samur M., Dimopoulos M., Kulakova M., Lam A., Hashim M. (2020). A large meta-analysis establishes the role of MRD negativity in long-term survival outcomes in patients with multiple myeloma. Blood Adv..

[52] Corre J., Mahtouk K., Attal M., Gadelorge M., Huynh A., Fleury-Cappellesso S., Danho C., Laharrague P., Klein B., Reme T. (2007). Bone marrow mesenchymal stem cells are abnormal in multiple myeloma. Leukemia.

[53] Todoerti K., Lisignoli G., Storti P., Agnelli L., Novara F., Manferdini C., Codeluppi K., Colla S., Crugnola M., Abeltino M. (2010). Distinct transcriptional profiles characterize bone microenvironment mesenchymal cells rather than osteoblasts in relationship with multiple myeloma bone disease. Exp. Hematol..

[54] Garderet L., Mazurier C., Chapel A., Ernou I., Boutin L., Holy X., Gorin N.C., Lopez M., Doucet C., Lataillade J.J. (2007). Mesenchymal stem cell abnormalities in patients with multiple myeloma. Leuk. Lymphoma.

[55] Lemaitre L., Hamaidia M., Descamps J.G., Do Souto Ferreira L., Joubert M.V., Gadelorge M., Avet-Loiseau H., Justo A., Reina N., Deschaseaux F. (2022). Toll-like receptor 4 selective inhibition in medullar microenvironment alters multiple myeloma cell growth. Blood Adv..

[56] Morohashi H., Miyawaki T., Nomura H., Kuno K., Murakami S., Matsushima K., Mukaida N. (1995). Expression of Both Types of Human Interleukin-8 Receptors on Mature Neutrophils, Monocytes, and Natural-Killer-Cells. J. Leukocyte Biol..

[57] Zhou Y., Zhou B., Pache L., Chang M., Khodabakhshi A.H., Tanaseichuk O., Benner C., Chanda S.K. (2019). Metascape provides a biologist-oriented resource for the analysis of systems-level datasets. Nat. Commun..

[58] Baumgart S.J., Najafova Z., Hossan T., Xie W., Nagarajan S., Kari V., Ditzel N., Kassem M., Johnsen S.A. (2017). CHD1 regulates cell fate determination by activation of differentiation-induced genes. Nucleic Acids Res..

[59] Wong A.H., Shin E.M., Tergaonkar V., Chng W.J. (2020). Targeting NF-kappaB Signaling for Multiple Myeloma. Cancers.

[60] De Jong M.M.E., Fokkema C., Papazian N., van Heusden T., Vermeulen M., Tahri S., Hoogenboezem R., van Duin M., van de Woestijne P., Langerak A. (2022). Stromal Cell-Activated Bone Marrow Neutrophils Provide BAFF in Newly Diagnosed and Treated Multiple Myeloma. Blood.

[61] Meads M.B., Hazlehurst L.A., Dalton W.S. (2008). The bone marrow microenvironment as a tumor sanctuary and contributor to drug resistance. Clin. Cancer Res..

[62] De Jong M., Fokkema C., Papazian N., van Heusden T., Vermeulen M., Hoogenboezem R., van Beek G., Tahri S., Sanders M.A., van de Woestijne P. (2023). An IL-1β driven neutrophil-stromal cell axis fosters a BAFF-rich microenvironment in multiple myeloma. bioRxiv.

[63] Holthof L.C., van der Schans J.J., Katsarou A., Poels R., Gelderloos A.T., Drent E., Van Hal-van Veen S.E., Li F.Z., Zweegman S., van de Donk N.W.C.J. (2021). Bone Marrow Mesenchymal Stromal Cells Can Render Multiple Myeloma Cells Resistant to Cytotoxic Machinery of CAR T Cells through Inhibition of Apoptosis. Clin. Cancer Res..

[64] Dhodapkar K.M., Cohen A.D., Kaushal A., Garfall A.L., Manalo R.J., Carr A.R., McCachren S.S., Stadtmauer E.A., Lacey S.F., Melenhorst J.J. (2022). Changes in Bone Marrow Tumor and Immune Cells Correlate with Durability of Remissions Following BCMA CAR T Therapy in Myeloma. Blood Cancer Discov..

