# Supplementary material for: Inflammatory Bone Marrow Mesenchymal Stem Cells in Multiple Myeloma: Transcriptional Signature and In Vitro Modeling
Source: Cancers (Basel). 2023 Oct 26;15(21):5148. doi: 10.3390/cancers15215148 (PMC10650304; doi:10.3390/cancers15215148)
Supplement: Supplementary file 1 [file cancers-15-05148-s001.zip › Figures S1-S5.pdf]

Supplemental Information

Figure S1

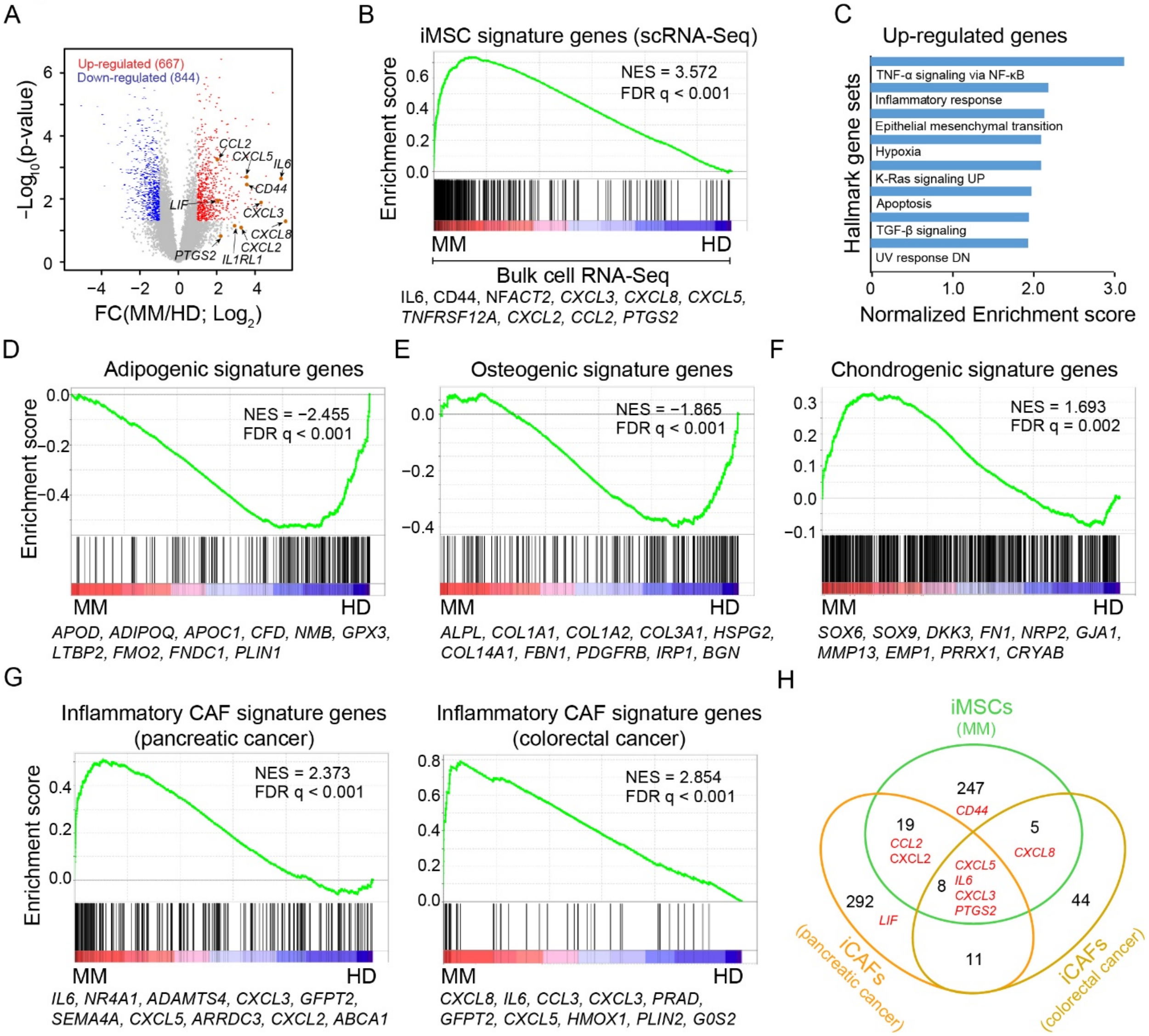

Supplementary Figure S1: Molecular signatures of iMSCs captured by bulk cell RNA-Seq for MM patients. (A) Volcano-plot displaying fold changes of gene expression and p-values from bulk-cell RNA-Seq analysis of BM MSCs to differentiate MM patients (MM) from healthy donors (HD). Red: up-regulated genes in MM. Blue: down-regulated genes. Gray: other expressed genes. Indicated by orange dots and arrow heads are examples of immune-related genes. (B) GSEA of expressed genes sorted by expression changes from high (red in the color spectrum) to low (blue) in BMSCs of MM as relative to HD (calculated from bulk cell RNA-Seq data) against iMSC signature genes defined for MM patients from scRNA-Seq analysis (vertical bars). Highlighted are selected leading genes related to inflammatory response. NES: normalized enrichment score. (C) Top hits of GSEA conducted as in panel B but against MSigDB hallmark gene sets. (D) GSEA like panel B but against adipogenic signature genes. Highlighted are representative leading genes enriched in adipocytes. (E) GSEA like panel B but against osteogenic signature genes. Highlighted are representative leading genes enriched in osteoblasts. (F) GSEA like panel B but against chondrogenic signature genes. Highlighted are representative leading genes enriched in chondrocytes. (G) GSEA like panel B but against iCAF signature genes defined for pancreatic cancer patients (left panel) or colorectal cancer patients (right panel). Highlighted are top ten leading genes. (H) Venn diagram comparing iMSC signature genes defined for MM patients to iCAF signature genes defined for pancreatic cancer patients and colorectal cancer patients. In red are examples of immune-related genes.

Figure S2

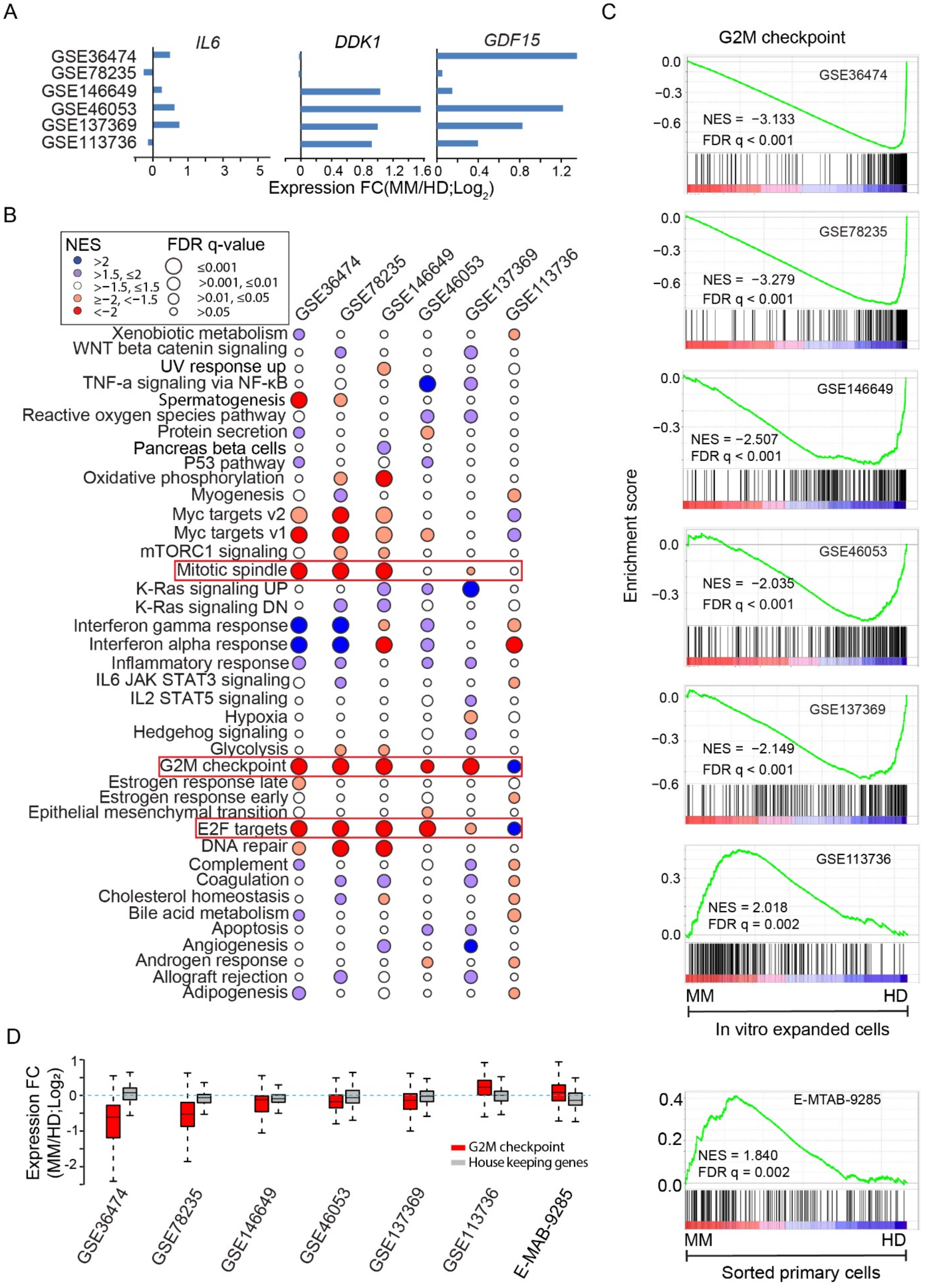

A
IL6
DDK1
GDF15
GSE36474
GSE78235
GSE146649
GSE46053
GSE137369
GSE113736
Expression FC(MM/HD;Log2)
B
NES
>2
>1.5, ≤2
>−1.5, ≤1.5
≥−2, <−1.5
<−2
FDR q-value
≤0.001
>0.001, ≤0.01
>0.01, ≤0.05
>0.05
Xenobiotic metabolism
WNT beta catenin signaling
UV response up
TNF-a signaling via NF-κB
Spermatogenesis
Reactive oxygen species pathway
Protein secretion
Pancreas beta cells
P53 pathway
Oxidative phosphorylation
Myogenesis
Myc targets v2
Myc targets v1
mTORC1 signaling
Mitotic spindle
K-Ras signaling UP
K-Ras signaling DN
Interferon gamma response
Interferon alpha response
Inflammatory response
IL6 JAK STAT3 signaling
IL2 STAT5 signaling
Hypoxia
Hedgehog signaling
Glycolysis
G2M checkpoint
Estrogen response late
Estrogen response early
Epithelial mesenchymal transition
E2F targets
DNA repair
Complement
Coagulation
Cholesterol homeostasis
Bile acid metabolism
Apoptosis
Angiogenesis
Androgen response
Allograft rejection
Adipogenesis
C
G2M checkpoint
Enrichment score
GSE36474
NES = −3.133
FDR q < 0.001
GSE78235
NES = −3.279
FDR q < 0.001
GSE146649
NES = −2.507
FDR q < 0.001
GSE46053
NES = −2.035
FDR q < 0.001
GSE137369
NES = −2.149
FDR q < 0.001
GSE113736
NES = 2.018
FDR q = 0.002
MM
HD
In vitro expanded cells
E-MTAB-9285
NES = 1.840
FDR q = 0.002
MM
HD
Sorted primary cells
D
Expression FC (MM/HD;Log2)
G2M checkpoint
House keeping genes
GSE36474
GSE78235
GSE146649
GSE46053
GSE137369
GSE113736
E-MAB-9285

Supplementary Figure S2: Down-regulation of proliferation-related pathways in MM MSCs expanded in vitro. (A) Expression fold-change of *IL6*, *DKK1*, and *GDF15* in BM MSCs in vitro expanded from MM patients (MM) as relative to those expanded from healthy donors (HD). Accession number for each study indicated on the left. (B) Bubble plot visualization of results from GSEA of expression changes contrasting MSCs in vitro expanded from MM to HD against MSigDB hallmark gene sets (rows) across different studies (columns). Color indicates an overall upregulation (blue) or downregulation (red) at gene set level. Circle size indicates the significance measured by FDR q-value. In red rectangle are three gene sets linked to cell proliferation. (C) GSEA of expressed genes sorted by expression changes of in vitro expanded MSCs (MM/HD) from high (red) to low (blue) against MSigDB hallmark gene set “G2M checkpoint” (vertical bars). NES: normalized enrichment score. (D) Boxplot visualization of expression fold-change (MM/HD) for genes from gene set “G2M checkpoint” or house-keeping genes across studies (rows).

Figure S3

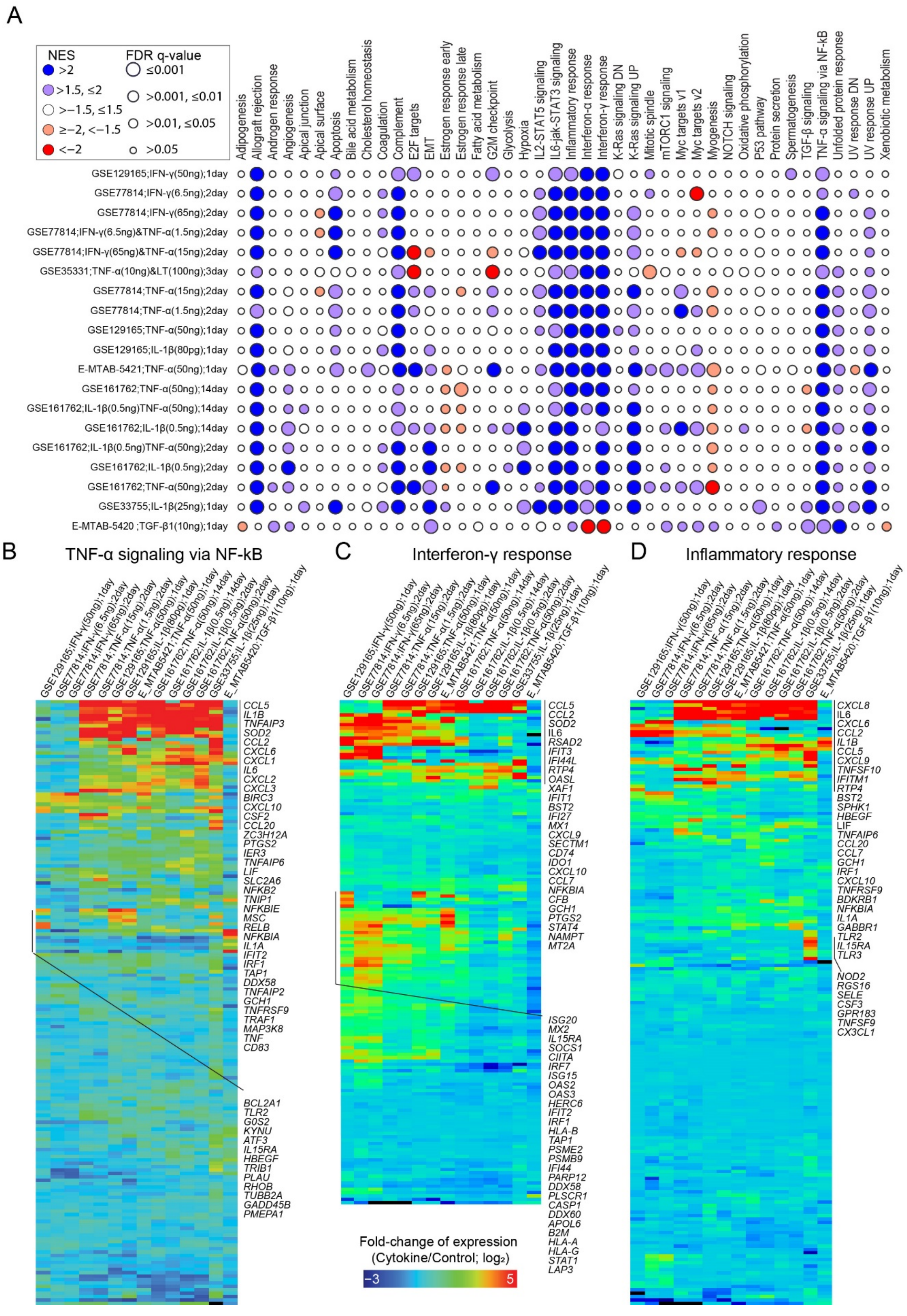
A
NES
>2
>1.5, ≤2
>−1.5, ≤1.5
≥−2, <−1.5
<−2
FDR q-value
≤0.001
>0.001, ≤0.01
>0.01, ≤0.05
>0.05
Adipogenesis
Allograft rejection
Androgen response
Angiogenesis
Apical junction
Apical surface
Apoptosis
Bile acid metabolism
Cholesterol homeostasis
Coagulation
Complement
E2F targets
EMT
Estrogen response early
Estrogen response late
Fatty acid metabolism
G2M checkpoint
Glycolysis
Hypoxia
IL2-STAT5 signaling
IL6-jak-STAT3 signaling
Inflammatory response
Interferon-α response
Interferon-γ response
K-Ras signaling DN
K-Ras signaling UP
Mitotic spindle
mTORC1 signaling
Myc targets v1
Myc targets v2
Myogenesis
NOTCH signaling
Oxidative phosphorylation
P53 pathway
Protein secretion
Spermatogenesis
TGF-β signaling
TNF-α signaling via NF-kB
Unfolded protein response
UV response DN
UV response UP
Xenobiotic metabolism
GSE129165;IFN-γ(50ng);1day
GSE77814;IFN-γ(6.5ng);2day
GSE77814;IFN-γ(65ng);2day
GSE77814;IFN-γ(6.5ng)&TNF-α(1.5ng);2day
GSE77814;IFN-γ(65ng)&TNF-α(15ng);2day
GSE35331;TNF-α(10ng)&LT(100ng);3day
GSE77814;TNF-α(15ng);2day
GSE77814;TNF-α(1.5ng);2day
GSE129165;TNF-α(50ng);1day
GSE129165;IL-1β(80pg);1day
E-MTAB-5421;TNF-α(50ng);1day
GSE161762;TNF-α(50ng);14day
GSE161762;IL-1β(0.5ng)TNF-α(50ng);14day
GSE161762;IL-1β(0.5ng);14day
GSE161762;IL-1β(0.5ng)TNF-α(50ng);2day
GSE161762;IL-1β(0.5ng);2day
GSE161762;TNF-α(50ng);2day
GSE33755;IL-1β(25ng);1day
E-MTAB-5420 ;TGF-β1(10ng);1day
B
TNF-α signaling via NF-kB
CCL5
IL1B
TNFAIP3
SOD2
CCL2
CXCL6
CXCL1
IL6
CXCL2
CXCL3
BIRC3
CXCL10
CSF2
CCL20
ZC3H12A
PTGS2
IER3
TNFAIP6
LIF
SLC2A6
NFKB2
TNIP1
NFKBIE
MSC
RELB
NFKBIA
IL1A
IFIT2
IRF1
TAP1
DDX58
TNFAIP2
GCH1
TNFRSF9
TRAF1
MAP3K8
TNF
CD83
BCL2A1
TLR2
G0S2
KYNU
ATF3
IL15RA
HBEGF
TRIB1
PLAU
RHOB
TUBB2A
GADD45B
PMEPA1
C
Interferon-γ response
CCL5
CCL2
SOD2
IL6
RSAD2
IFIT3
IFI44L
RTP4
OASL
XAF1
IFIT1
BST2
IFI27
MX1
CXCL9
SECTM1
CD74
IDO1
CXCL10
CCL7
NFKBIA
CFB
GCH1
PTGS2
STAT4
NAMPT
MT2A
ISG20
MX2
IL15RA
SOCS1
CIITA
IRF7
ISG15
OAS2
OAS3
HERC6
IFIT2
IRF1
HLA-B
TAP1
PSME2
PSMB9
IFI44
PARP12
DDX58
PLSCR1
CASP1
DDX60
APOL6
B2M
HLA-A
HLA-G
STAT1
LAP3
Fold-change of expression
(Cytokine/Control; log₂)
−3
5
D
Inflammatory response
CXCL8
IL6
CXCL6
CCL2
IL1B
CCL5
CXCL9
TNFSF10
IFITM1
RTP4
BST2
SPHK1
HBEGF
LIF
TNFAIP6
CCL20
CCL7
GCH1
IRF1
CXCL10
TNFRSF9
BDKRB1
NFKBIA
IL1A
GABBR1
TLR2
IL15RA
TLR3
NOD2
RGS16
SELE
CSF3
GPR183
TNFSF9
CX3CL1

Supplementary Figure S3: Expression activation of TNF-α signaling pathway in MM MSCs by cytokines. (A) Bubble plot visualization of results from GSEA of expression changes induced by cytokine stimulations (rows) in BM MSCs against MSigDB hallmark gene sets (columns). Color indicates upregulation (blue) or downregulation (red) at gene set level. Circle size indicates significance (FDR q-value). (B) Heat map visualization of expression changes induced by mono-treatment of cytokines including IFN-γ, IL1-β, TNA-α, and TGF-β1 for gene set "TNF-α signaling through NF-κB". Top upregulated genes were indicated. (C) Like panel B, but for gene set "IFN-γ response". (D) Like panel B, but for gene set "inflammatory response".

Figure S4

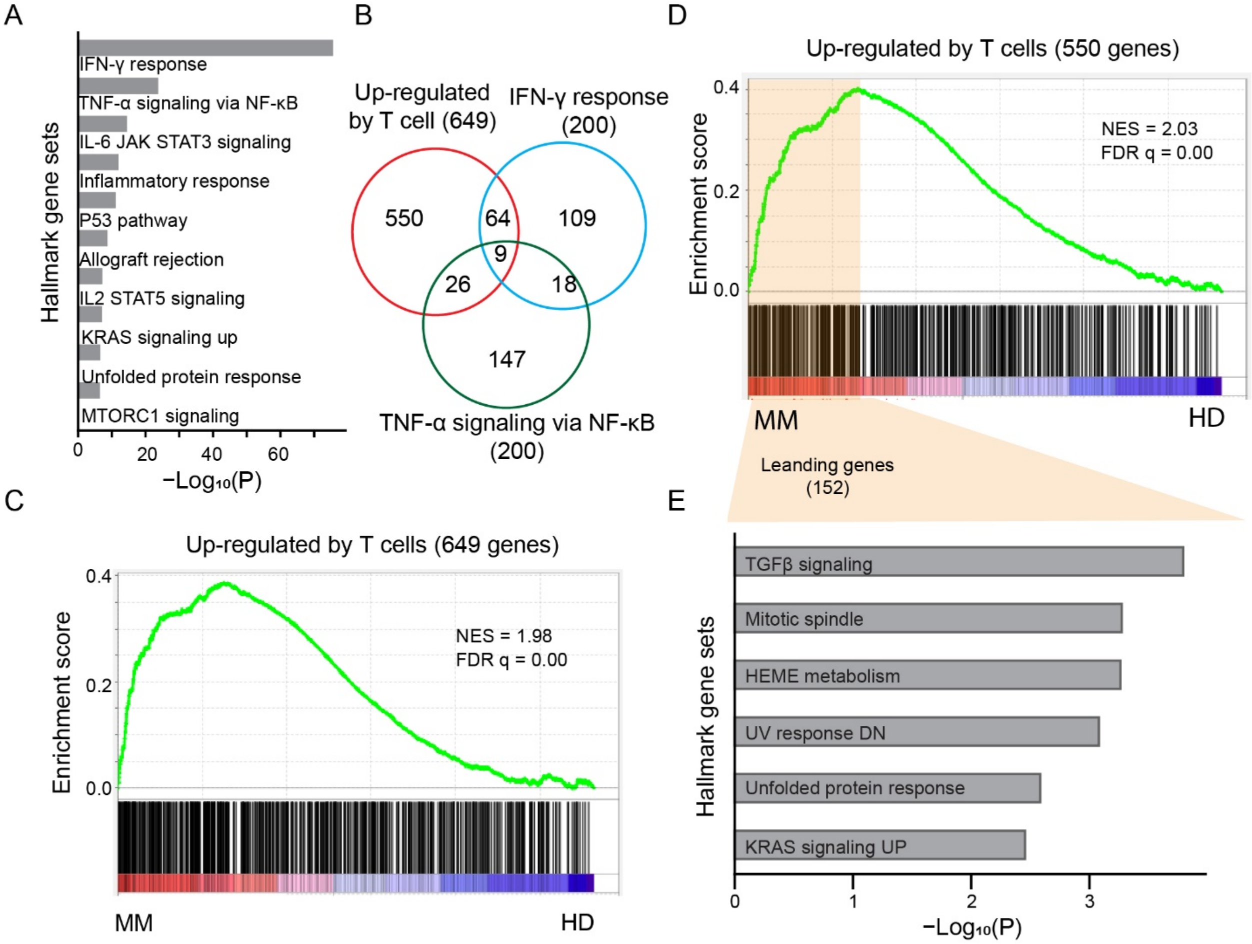

Supplementary Figure S4: Expression activation of iMSC signature genes by activated T cells. (A) Metascape enrichment analysis for genes upregulated by activated T cells in BM MSCs against MSigDB hallmark gene sets. (B) Venn diagram for genes upregulated by activated T cells and genes annotated in “IFN-γ response” and “TNF-α signaling via NF-κB”. (C) GSEA of expressed genes sorted by expression changes from high (red in the color spectrum) to low (blue) in BMSCs of MM as relative to HD (calculated from bulk cell RNA-Seq data) against the 649 genes upregulated by activated T cells (vertical bars). NES: normalized enrichment score. (D) Similar to panel C, but against genes upregulated by activated T cells excluding those from “IFN-γ response” or “TNF-α signaling via NF-κB”. (E) Metascape enrichment analysis for leading genes defined from panel D against MSigDB hallmark gene sets.

Figure S5

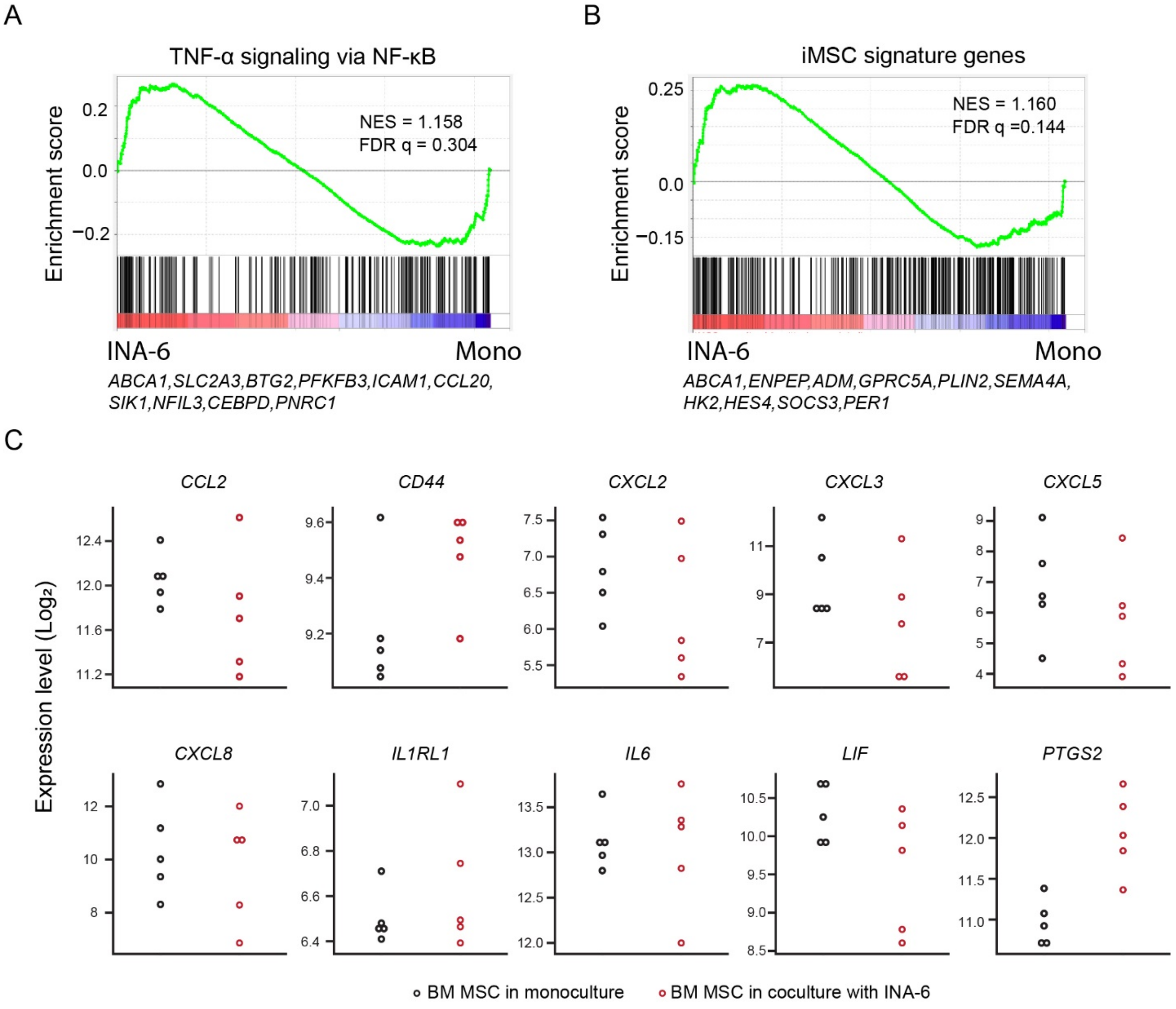

Supplementary Figure S5: INA-6 failed to active the expression of iMSC signature genes. (A) GSEA of expressed genes sorted by expression changes induced by INA-6 (as compared to monoculture; Mono) in BM MSCs from high (left side) to low (right side) against MSigDB hallmark gene set “TNF-a signaling via NF-κB”. NES: normalized enrichment score. (B) Like panel A, but against iMSC signature genes. (C) Dot plots for the expression of immune-related genes in BM MSCs cocultured with INA-6 or in monoculture.
